# Collective electronic excitations in the ultra violet regime in 2-D and 1-D carbon nanostructures achieved by the addition of foreign atoms

**DOI:** 10.1038/srep27090

**Published:** 2016-06-07

**Authors:** U. Bangert, W. Pierce, C. Boothroyd, C.-T. Pan, R. Gwilliam

**Affiliations:** 1 Department of Physics and Energy, University of Limerick, Limerick, Ireland; 2 School of Materials, The University of Manchester, Manchester M13 9PL, United Kingdom; 3 Ernst Ruska-Centre for Microscopy and Spectroscopy with Electrons and Peter Gruenberg Institute Juelich Research Centre, D-52425 Juelich, Germany; 4 School of Materials Science and Engineering, Nanyang Technological University, 50 Nanyang Avenue, Singapore 639798.; 5 Advanced Technology Institute, University of Surrey, Guildford GU2 7XH, United Kingdom

## Abstract

Plasmons in the visible/UV energy regime have attracted great attention, especially in nano-materials, with regards to applications in opto-electronics and light harvesting; tailored enhancement of such plasmons is of particular interest for prospects in nano-plasmonics. This work demonstrates that it is possible, by adequate doping, to create excitations in the visible/UV regime in nano-carbon materials, i.e., carbon nanotubes and graphene, with choice of suitable ad-atoms and dopants, which are introduced directly into the lattice by low energy ion implantation or added via deposition by evaporation. Investigations as to whether these excitations are of collective nature, i.e., have plasmonic character, are carried out via DFT calculations and experiment-based extraction of the dielectric function. They give evidence of collective excitation behaviour for a number of the introduced impurity species, including K, Ag, B, N, and Pd. It is furthermore demonstrated that such excitations can be concentrated at nano-features, e.g., along nano-holes in graphene through metal atoms adhering to the edges of these holes.

Research in plasmonics has dramatically increased in recent years, with a number of suggested, possible applications, including biosensors, solar cells and sub-wavelength optics^1^,^2^,^3^,^4^,^5^. Plasmon research in graphitic carbon (nano) materials has originally been carried out on the graphitic π-plasmon^6^,^7^, which has energies far beyond the visible and also the UV range. More recently coupling of light to plasmons in the visible/UV energy regime has become a field of great interest with many applications for light guiding and enhancement/confinement at nano-features. Carbon nanotubes (CNTs), for example, due to their 1-D nature, are prospective candidates for plasmon waveguides. One of the incentives is to use them in photovoltaics to harvest light by coupling to plasmons, which are then guided to be re-emitted at other points, or to reach p-n junctions for decay into e-h pairs and hence current production^2^,^4^,^5^. The prominent graphitic π-plasmon has energies far in the UV, so is not apt for this process. By adequate doping it might be possible to create plasmons in the visible regime in graphitic structures, with suitable dopants being introduced in their pure form by low energy ion implantation.

Since graphene has been ‘put on the map’, apart from the attention it has received due to its unusual electronic properties, such as high carrier mobility^8^,^9^ and ballistic transport^9^, considerable interest has arisen in its plasmons. Attention has so far focussed on the intraband charge carrier plasmons which lie in the Terahertz or infra-red regions and which show potential for applications in THz plasmonic devices, e.g., as optoelectronic components and light sensors. These plasmons originate from the collective oscillation of the free charge carriers^10^,^11^ and in graphene they occur at energies in the far-infrared region^12^,^13^; their distribution in nanostructures has been revealed by infrared nano-imaging^14^,^15^. However, there is also interest in the optical region through to the UV^3^, including tuneable optical absorption^16^,^17^ Whilst intense research efforts have been invested into graphene plasmonics in the infra-red and Terahertz range, far less research has been directed towards the visible and UV absorption range. It has been demonstrated that due to its π-electron system graphene also has inter-band plasmon features, which are similar to plasmons in CNTs. These occur at higher energies around 4.6 eV and 14.5 eV and arise due to transitions between saddle points in the π-bands at the M-point in the k-space diagram, enhanced by Van Hove singularities^18^,^19^. The existence of plasmons in the high UV (~4–5 eV) regime in graphene, however, has been much disputed^20^, it is argued that, due to the non-zero crossing of the dielectric function (a zero-crossing is the criterion of collective, i.e., plasmon excitations), features previously deemed as graphitic π-plasmons are either heavily damped, or coupled to single-particle interband transitions. Regardless, however, of whether a feature is a plasmon, its potential for local enhancement of light absorption/ emission is of interest, e.g., for development of new photonics and sunlight harvesting devices.

Here we present electron energy loss studies in the 2–4 eV range of dosed and doped CNTs and graphene. We investigate spectral features, spanning the range from visible (green) to far UV frequencies, introduced by doping of CNTs with alkali and earth alkali metals Ag and B, and doping or dosing of graphene with N and transition metals. To achieve doping we carry out low-energy implantation, which enables direct impurity incorporation and modification of the electronic structure.

As mentioned above, few-layer and single wall nanotubes (SWNTs) and graphene have a pronounced absorption feature at ~4.6 eV; in the case of SWNTs this is deemed to be a collective interband excitation, the π-plasmon. As also mentioned above, the existence of plasmons in graphene at these energies has been disputed. Here we show via electron energy loss spectroscopy (EELS) studies that there is a new, unambiguous absorption component at ~3 eV, whose origin is still under discussion: it could be a collective mode component or a single particle excitation or both. Assessing energy losses in the low loss/optical regime via EELS in a transmission electron microscope (TEM) is usually restricted by the energy spread of the electron beam, although monochromation and post-acquisition deconvolution methods can push the limits to which spectral features can be resolved down to 1 eV, enabling observations of variations in the extent and structure of the 3-eV feature. These techniques reveal that the peak at 3 eV varies in intensity and structure when dosing/doping has been applied; there is usually a significant increase in intensity with increasing doping level. We provide evidence that this excitation has indeed plasmonic character by the revelation of a zero-crossing of the extracted dielectric function. Furthermore, a highly localised enhancement of this feature has been observed in graphene, where strong spatial inhomogeneities of impurity atom distributions, e.g., after dosing with metals, occurred. Since metal atoms move preferentially to edges in graphene sheets, this might be a promising way to ‘nano-sculpt’ light emission.

## Experimental

### Sample preparation

CNTs and graphene were produced via chemical vapour deposition (CVD). Graphene was produced via CVD of CH_4_ and H_2_ precursor gasses on a copper foil substrate^21^. CNT suspensions were dripped and dried on holey carbon supported copper grids for transmission electron microscopy (TEM) investigations. The graphene was transferred onto copper TEM grids coated with a carbon support film containing regularly spaced holes (Quantifoil), using the ‘wet transfer’ method^22^. Freestanding graphene produced using this method was mainly monolayer, with occasional bilayer or multilayer regions. As with all graphene, residual hydrocarbon deposits were seen on the samples.

Results presented in this paper are obtained on CNTs and graphene treated/modified by adding foreign atom species as listed in Table 1. This table summarises the elements and their doses as well as the way in which they were introduced and the instrument used for their investigation. The table also refers to the figures in which the results are displayed.

CNTs were doped directly by introducing alkali and earth alkali metals (e.g. K, Na and Ba) as well as Ag, B and N. Results of implantation with B and N have been presented in^23^; here we elaborate further on these results with regards to new low loss features. The elements were introduced into CNTs via ion implantation at ~200 eV at the Surrey University Ion Beam Centre (B-implantation under the same conditions was additionally carried out at the Salford University Low Energy Ion Implanter in some of the samples). The implantation energy was chosen to ensure minimal damage and at the same time uniform doping over the nanotube volume as there are multiple carbon sheets through which the ion beam has to proceed, due to the existence of multiple nanotube walls (in few- or multiple-wall nanotubes (MWNTs)) or bundles of tubes (SWNT bundles) as well as tube curvature, resulting in the ion beam being incident at multiple and varied (often shallow) angles.

Substitutional doping of graphene with N and B was achieved using ultra-low-energy ion implantation as demonstrated previously^24^. Here we include new low loss features arising from N-doping. Energies of ~25 eV were chosen and implantation was carried out with the University of Göttingen mass selected ion beam deposition system^25^,^26^ directly into above described free standing CVD graphene at doses between 10^14^ and 10^15^ cm^−2^. The exact retained dose is hard to determine via HRTEM methods due to the large number of images needed to gather adequate statistics. In support of the ultra-low energy ion-implantation experiments simulations of the implantation process in graphene were previously performed in Göttingen using the Monte Carlo program SDTrimSP^25^,^27^. Although recent studies have indicated that Monte-Carlo codes such as TRIM may be insufficient when calculating ion impacts in graphene^27^, the calculations should provide a good first approximation; in the case of N they suggested a doping level of 1.5 to 5 at%^24^,^25^. HRTEM images indicate nitrogen doping levels ranging from 0.1 at% to 1 at%^25^, and higher (few %) for alkali-ions in CNTs.

Metals (Pd and Ti) were deposited onto the graphene using electron-beam or thermal evaporation. An evaporation rate of 0.1 nm/s was used and the amount of metal deposited varied between 0.1 nm and 0.3 nm.

### Data acquisition

**Figure 1:**
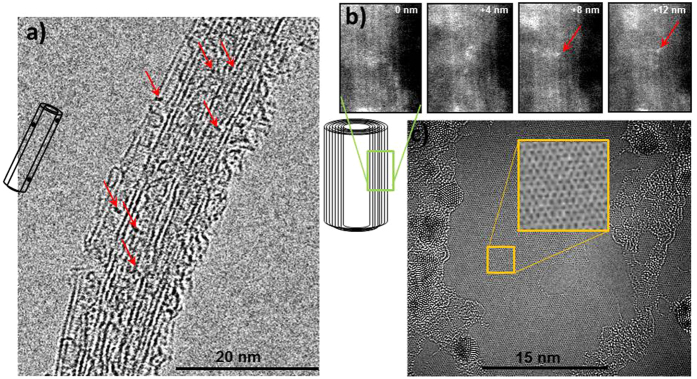
(**a**) HREM image of a K- implanted DWNT bundle; dark dots (some are arrowed) are K-atoms, seen in many cases between the graphene sheets (see sketch), indicating the intercalated state, (**b**) HAADF images from a through-focal series of a wall of a MWNT implanted with Ag ions: bright dots are intercalated Ag atoms sitting at different depths; e.g., the arrowed atom is in focus at defocus values between +8 and +12 nm, (**c)** HREM image of graphene onto which a Pd film of 0.1–0.3 nm thickness was evaporated. The Pd forms nano-crystals, which sit in the contamination (in the middle there is a clean graphene patch). *For details about samples and measurement see* Table 1.

**Figure 2:**
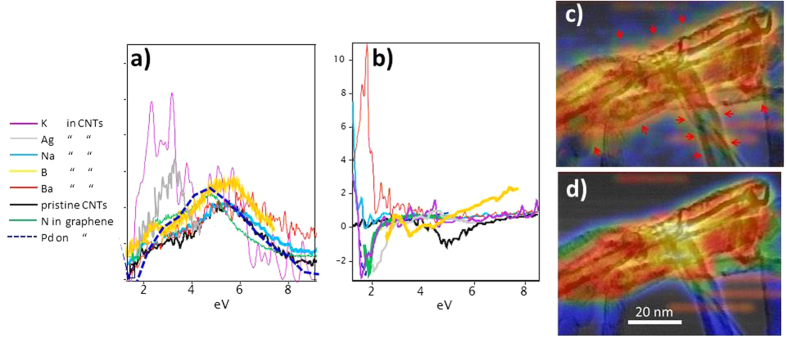
(**a**) Low loss EEL spectra of DWNTs and SWNT bundles implanted with alkali (K, Na), earth-alkali (Ba), transition metals (Ag) and B; also shown is a spectrum of N-implanted (substitutionally doped^24^) and of Pd-dosed graphene (see image in Fig. 1c). The latter spectrum was obtained from the edge of a hole in pristine graphene decorated with Pd atoms (more info is presented in Fig. 4, top row, and Fig. 5b,c). The spectrum intensity is normalised to the feature at ~5 eV (π-plasmon). (**b**) Re*ε*_*m*_of the dielectric functions extracted via Kraemers-Kroenig analysis from the experimental spectra in (**a**), smoothed with a low pass filter with w = 0.2 eV; the zero crossing with positive gradient at 2.5–3.5 eV for K-, Ag-, B-, N- and Pd doped/dosed samples is a sign of collective charge carrier behaviour. (**c,d**) EEL signal maps of a DWNT and a few-layer B-implanted CNT agglomerate in energy windows of 2–3.5 eV (new plasmon feature) and 4.7–6.5 eV (π-bulk plasmon), respectively, after background subtraction. The maps are extracted from an EEL spectrum image. The EEL map is overlaid on the Bright Field STEM image of the tubes (black shapes). Both EEL images are bIurred to the same extent (5 pixels) and maximum and minimum signals adjusted to the same level on the temperature scale. Arrows in (**c**) mark positions that indicate that the lower energy signal is less localised than the π-bulk plasmon in (**d**) and more concentrated on the surface of the tubes, extending into the vacuum. (The horizontal orange lines in both images are scan related noise.) *For details about samples and measurement see* Table 1.

**Figure 3:**
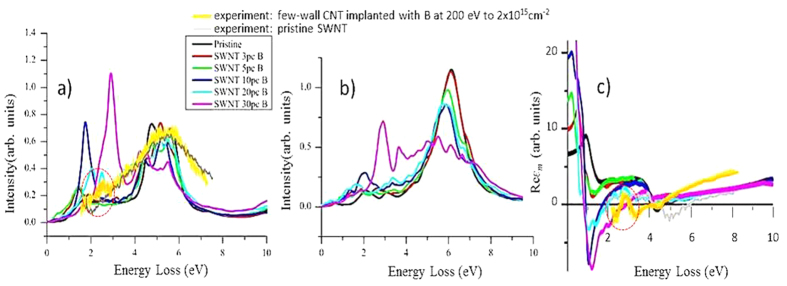
Calculated electron energy loss spectra of a (10,10) SWNT bundle, B-doped via ion implantation to various B concentrations, in (**a**) the out-of-plane and (**b**) the in-plane direction; (**c**) calculated out-of-plane component of the real part of the dielectric function. The calculations were performed using Wien2k. Also overlaid in (**a**,**c**) are the experimental spectrum and the extracted real part, Re*ε,* of a heavily B-doped (orange) and a pristine (grey) carbon nanotube (also shown in Fig. 2). *For details about samples and measurement see* Table 1.

Measurements on the Ag-implanted CNTs (Fig. 1b), were carried out at the Daresbury SuperSTEM^23^, and on the B-implanted CNTs (Fig. 2) in the Liverpool NorthWest STEM. Electron energy loss spectroscopy in scanning mode tends to be the preferred way to acquire energy loss data. Data cubes obtained from Spectrum Images (SIs) contain an entire spectrum in each defined image pixel. Evaluation of such data is relatively straight-forward, as it is ‘spectrum controlled’, hence post-acquisition energy alignment and intensity calibration of specific loss features to those in other experimental or calculated spectra (e.g.Fig. 3) can be easily carried out without further calibration measurements. The acquisition of SIs is, however, slow, and due to the high beam currents of the focussed probe, changes in sample morphology are often induced, even with short acquisition times, especially in 2-D and 1-D materials. Hence it is mostly restricted to small areas. Energy filtered imaging (EFTEM) on the other hand provides fast acquisition of large-area energy loss images, making it is less destructive then spectrum imaging, due to the lower beam currents, at the same time enabling sequential images (data cubes) to be taken with energy steps in the meV range. It requires, however, correction of non-isochromaticity and afterglow effects, procedures for which are explained in the results section.

**Figure 4:**
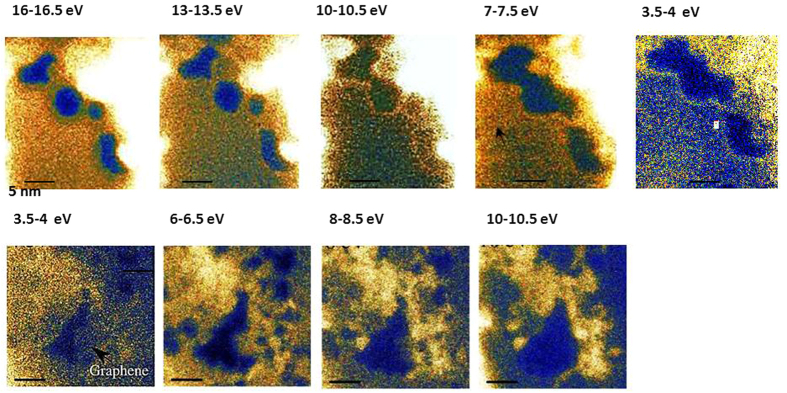
Top row, left to right: ZLP subtracted images extracted from an EFTEM cube of Pd dosed graphene showing changes in the sample morphology and also in the image intensity distribution during recording. Image slices are recorded at decreasing loss energies to minimise afterglow effects form high intensities in the lower loss region. The dark blue regions seen in the image at 16.5 eV are holes, which appear and grow after repeated exposure to the electron beam during recording (to build up the data cube), and eventually, at around 10 eV, merge into a larger hole, enlarging further. The very bright regions arise due to Pd crystals (an HREM image of a near-by area of graphene dotted with Pd crystals, which has not undergone prolonged beam exposure, is shown in Fig. 1c). Note the intensity enhancement along the hole’s edge in the image slices of 10–10.5 and 3.5–4 eV. Bottom row, left to right: ZLP subtracted images extracted from an EFTEM cube of pristine graphene, this time recorded at increasing loss energies. The hole in the 3.5–4 eV energy slice increases this time more rapidly, under continued recording. *For details about samples and measurement see* Table 1.

All energy filtered TEM (EFTEM) imaging (Fig. 4) and energy loss spectroscopy (EELS) as well as high resolution bright field electron microscopy (HREM; Fig. 1c) was carried out at the Ernst-Ruska Centre, Jülich, on an FEI Titan 50–300 Pico triple aberration corrected and monochromated TEM operated at 80 keV. The microscope is equipped with a Gatan UltraScan 4000 UHS CCD with 4k × 4k pixels. EFTEM images and EELS were acquired using a Gatan Quantum ERS image filter with fully 2^nd^ and 3^rd^ order and partially 4^th^ order corrected prisms. EFTEM images were acquired with a slit width of between 0.4 and 1 eV and with energy step sizes of between 0.1 eV and 0.5 eV. Low loss EFTEM data cubes were then collected, consisting of a stack of EFTEM images (with the x- and y-directions defining the image coordinates and the z-direction acting as energy axis) spanning an energy range from typically −2 eV to 18 eV. EEL spectra were (i) extracted from such EFTEM data cubes or (ii) directly acquired in scanning (STEM) mode. Monochromation of the electron beam enabled the FWHM of the zero-loss peak to be reduced to around 0.1 eV.

### Data evaluation

In order to access data below 5 eV it is necessary to remove the zero-loss peak (ZLP). This was done in Gatan Digital Micrograph (DM) by fitting a power-law background to the ZLP using a vacuum spectrum (obtained far from the sample) as a guide. An alternative approach, using the reflected-tail function in DM, was used for the EFTEM images recorded on the monochromated Titan Pico. This method assumes that the ZLP is symmetric. This is not strictly true for FEG sources, where the ZLP has an extended tail towards higher energies; however, monochromation has been shown to reduce the effect of this tail^28^. In some cases (on spectra obtained from metal- and B-doped CNTs) Richardson Lucy (RL) deconvolution was applied, bringing the spectral information down to 1 eV. The RL algorithm is a maximum-likelihood Baysian deconvolution method that can be used to sharpen low-loss peaks and to remove the ZLP [e.g.^29^, p. 242].

Calculations and modelling of low loss EEL spectra within the frame of the dielectric theory in order to support the measured EEL spectra are referred to in the Supplementary Information chapter. Such modelling entailed calculations of the energy loss function and complex dielectric function of graphene and SWNT systems using the Wien2k DFT code^30^. The latter helps investigations of whether an excitation is of collective (plasmon) nature. In order to validate the calculations the dielectric function was also extracted from experimental EEL spectra using the Kramers-Kroenig Analysis programme in Digital Micrograph (a full description of the analysis method can be found in the Gatan Documentation, EELS Analysis, Copyright 2012 Gatan Inc.). Kramers-Kroenig analysis enables the energy dependence of the real and imaginary parts of the dielectric function of the specimen to be calculated from the low-loss single scattering distribution [e.g.^29^, pp. 256–262]. The real and imaginary parts of the dielectric function provide a reasonably complete local description of the electronic and optical properties of the material under investigation, enabling optical properties such as the absorption coefficient and the reflectivity to be calculated. The imaginary part of the dielectric function may also be linked to the optical joint density of states for low-momentum transfers, allowing comparison with optical measurements.

## Results and Disucussion

### Effect of incorporated dopant atoms on the low loss specta of nano-carbons

#### Experimental observations

In the following we will give evidence of new excitations with plasmonic character, induced by impurities that are incorporated into or between lattice planes of sheet-like nano-carbons. In order to investigate/consolidate dopant-introduced optical/near-UV plasmon behaviour in sheet-like carbon nanostructures, K, Na, Ba, Ag, N and B were introduced via low-energy ion implantation into few-walled carbon CNTs, primarily SWNTs and DWNTs, succeeded by EELS investigations. A spectrum of N-implanted graphene is included in the results shown in the following, together with one of Pd-dosed graphene, to demonstrate the similarity of doping effects in sheet-like carbon nanostructures. Figure 1a shows an HREM image of a double wall CNT bundle implanted with K. The dark dots (some arrowed) under the present focussing conditions correspond to heavier elements, i.e., K-atoms. None of these contrast features can be observed in HREM images of pristine CNTs. The image shows furthermore that rather uniform doping with K-atoms has been achieved; the latter are often seen lining the dark stripes, which are the projections of the carbon lattice planes forming the CNT cylinder, suggesting that K is intercalated. Figure 1b shows a high angle annular dark field (HAADF) image of part of a wall of a multiple-sheet CNT, implanted with Ag. Here the projections of the cylindrical carbon sheets are bright lines, and the bright dots are intercalated Ag atoms coming in and out of focus in the through-focal series, from which the four images in Fig. 1b are extracted. This means that the Ag atoms are positioned at different depths in the wall; the arrowed atom, for example, is in focus at defocus values between +8 and +12 nm. It sits between two lines (carbon sheets), hence is intercalated. It can also be seen to be moving by slight amounts up and down between the lines in different images of the series, further supporting the suggestion that it is intercalated. Again, no such atomic-scale contrast features are observed in pristine CNTs. EEL low loss spectra of K-, Na-, Ag- and Ba-implanted CNTs are also shown in Fig. 2a. In order to assess EEL absorption features down to 1 eV, the spectra have undergone seven iterations of Richardson Lucy deconvolution using a vacuum zero loss peak (ZLP) obtained under identical conditions. Background fitting and subtraction was carried out in the same energy regime (0.5–1.2 eV) for all spectra. The spectra in Fig. 2a reveal that in these nanotubes Na and Ba (light blue and red curves) do not give rise to significant extra features and show near-identical spectra to pristine CNTs (black curve). Interestingly, the K-implants induce a very strong signal at energies 2–3.5 eV (purple curve), the nature of which, according to the zero-crossing of real part of the dielectric function, Re*ε*_*m*_, extracted by Kramers-Kronig analysis in Fig. 2b, is a plasmon excitation. The reason for the pronounced plasmon at 2–3 eV in the K-implanted sample requires further investigation. The evidence that K is present in the intercalated state in the CNT in Fig. 1a with a rather uniform distribution and high concentration is a likely explanation. HREM images of Na- and Ba- implanted CNTs, on the contrary, did not clearly reveal the dopants as intercalants, nor did they disclose a substantial dose or uniform distribution. A strong absorption feature at a slightly higher energy (grey curve) is furthermore observed for Ag implants. Again, their intercalant nature and significant retention can be directly observed (Fig. 1b). In the case of both, K- and Ag-implants, Re*ε*_*m*_ crosses zero at the energy of the maximum of the absorption peak. B-implants, although their presence in CNTs cannot easily be revealed in HREM or HAADF images due to B’s low atomic number, also introduce a distinct absorption feature at a slightly lower energy than K- and Ag-implants (orange curve), again with a zero crossing of Re*ε*_*m*_ at that energy. It should be noted that a non-zero Re*ε*_*m*_ does not necessarily imply non-collective nature, but can indicate a heavily damped plasmon^31^,^32^,^33^,^34^.

Doping of C-nanostructures with N and B is of significant interest for bandstructure engineering, similar to the case of doping Si with goup V- and III-elements. Furthermore, as reported earlier^23^ graphene has been successfully N-doped via low energy ion implantation with the dopants populating substitutional sites. Here we show first experimental results of new features in low loss spectra of N-implanted graphene, at a similar energy as the feature in B-doped CNTs. Figure 2a (green curve) shows a ZLP-subtracted experimental low loss spectrum of N-doped graphene, exhibiting a pronounced enhancement in the low loss spectrum at ~3 eV. Spectra of pristine CNTs (black curve in Fig. 2a) and similarly, of pristine graphene (black curve in Supplementary Fig. 1a) only exhibit a plateau in this region, in good agreement with the calculations in Supplementary Fig. 1b,c, bottom curve (a). The green curve in Fig. 2b is the Re*ε*_*m*_extracted via Kraemers-Kroenig analysis from the experimental spectrum for N-doped graphene (green curve in λ). There is a zero crossing of Re*ε*_*m*_ at around 3 eV, at the energy of the new absorption feature. As in the case of the CNTs this feature is very likely to represent a new plasmon. N-doping of single- and few-wall CNTs (not shown here) introduces a similar feature at ~3 eV, so it can be assumed that N-doping in CNTs induces similar electronic structure modifications as in graphene. Detailed measurements of graphene doped at various levels with N and also B, supported by calculations of electron energy loss spectra, are presented in a separate publication. Pd-dosing of graphene induces features similar to that of N-doped graphene (blue dashed curve in Fig. 2a); it also exhibits a similar Re*ε*_*m*_ with zero-crossing at the same energy (blue dashed curve in Fig. 2b); likewise does Ti introduce a strong absorption feature at ~3 eV (see Supplementary Fig. 1a).

Regarding spatial confinement and extent of the plasmonic features introduced by impurities, Fig. 2c,d show the EEL intensity distribution of a double- and few-layer B-implanted CNT agglomerate at 2–3.5 eV, the presumed new plasmon energy, and at 4.7–6.5 eV, the π-plasmon energy, respectively. The intensity maps are overlaid on the image of the CNTs agglomerate. The shapes of the latter can be recognised by the dark outlines. The signal in both energy regions is enhanced between and at the crossing of tubes. The arrows in Fig. 2c point to positions, namely along the tube surfaces, where the lower energy signal is less localised than the π-bulk plasmon signal in Fig. 2d), and also higher in intensity, which furthermore extends into the vacuum. This agrees with the new feature being a de-localised surface plasmon.

#### Modelling of low loss spectra

B-doping is of significant interest for mono- and few-layer carbon nanostructures. Like N-doping, substitutional B-doping, applied to achieve Fermi level shifts and donor- or acceptor-type behaviour, might introduce new UV/vis features. We have previously established^23^ that low-energy B-implantation into CNTs results in substitutional doping (previous results have shown that this is also the case for graphene^35^). In the following we support experimental results concerning the low loss feature induced by B by WIEN2K spectrum calculations. Figure 3a,b show out-of-plane and in-plane calculated spectra for SWNTs broadened by using a Lorentzian function with a gamma of 0.1 eV. EELS measurements excite both in-plane and out-of-plane modes^18^. Experimental spectra are hence a combination of the perpendicular and parallel components shown in the simulation. The relatively small broadening was chosen as a compromise to smooth the many spikes resulting from the calculations but at the same time, reveal as many changes as possible to the spectra of doped structures. The experimental spectrum of B-doped CNTs (orange, only overlaid on the in-plane calculations in Fig. 3a) resembles most closely the one of 20% B-doping level (light blue), exhibiting a small peak, sitting on the rise of the π-plasmon (encircled) in the 2–2.5 eV region. Neither the experimental (grey) nor the calculated (black) spectrum in Fig. 3a of pristine CNTs exhibit this peak. There is also a clear zero-crossing of the orange curve at ~2.5 eV in Fig. 3c, in line with this feature being a plasmon. The main experimental features for the doped and undoped case are reflected in the simulations, giving confidence in the calculated data.

So, calculations for nanotubes show that B- and N-doping introduces a new peak occurring at energies below the π plasmon. Here we only show spectra for B-doped SWNTs; calculations for N-doped CNTs are shown in the Supplementary Information (Supplementary Fig. 4). In the calculations this peak shifts in energy upwards when increasing the doping level, offering a possible route to tailoring plasmonic materials for the visible spectrum. This trend was previously reported for boron doped carbon nanotubes^23^. The observations supported by calculations suggest that all sp_2_ carbon materials behave similarly under doping. The precise origin of the pre-π- peak is not known. Figure 3c shows Re*ε*_*m*_ of the calculated spectra in Fig. 3a. For B-doping levels ≥10% Re*ε*_*m*_shows a distinct zero-crossing at 2–2.5 eV, in line with collective excitation behaviour. The zero approach and crossing of the dielectric functions of the experimental spectra for doped CNTs in this regime (orange for B-doping Figs 2b and 3b, purple for K-doping Fig. 2b) and also for doped/dosed graphene (green/ blue for N-doping/Pd-dosing in Fig. 2b), affirm the collective nature of this excitation. The likely cause is a doping-induced change in the Fermi level.

Occurrence of additional, new absorption peaks below the π-plasmon in alkali-doped SWNTs, where the doping resulted in intercalation of alkali atoms between the SWNTs, were observed by Liu *et al*.^36^. They are ascribed to a new charge carrier plasmon, created by ionic transfer and not by hybridisation of energy bands. Thus the π-plasmon is not affected by the new, additional charge carrier plasmon with energies starting above 1 eV and shifting upwards with increasing concentration of the intercalant. In contrast a new peak due to Ba intercalation was ascribed to hybridisation^37^. Here lattice distortion due to the intercalant (expansion of wall distance) causes bandstructure modification: the π-plasmon diminishes with increasing Ba-concentration and the energy of the new plasmon peak increases to 3 eV. In our case of double-wall or few-wall CNT bundles, the intercalation occurs significantly between the atomic sheets within the walls of individual tubes rather than between tubes. HREM images show no distortion of the lattice and the new plasmon peak in all cases occurs as shoulder (of varying strength depending on the dopant) of the otherwise unaffected π-plasmon, suggesting that the new feature is a charge carrier plasmon, similar to the one introduced by alkali intercalants in the SWNT bundles in^36^ above. We note that the ~3 eV feature in metal dosed graphene seems to be consistent with that observed in alkali- and Ag-dosed CNTs. Furthermore do the ~3 eV features in B- doped CNTs and N-doped CNTs and graphene (all by ion implantation) show similar behaviour, in terms of the low loss spectrum and the R*ε*_*m*_, suggesting the nature of this feature to be a charge carrier plasmon in the carbon material.

In support of the experimental observations in graphene, calculations for frequently observed metal-ad-atom configurations^38^ of Pd and also Ti on graphene (Supplementary Fig. 1a,b, respectively) have been carried out, which show intensity enhancement at ~3 eV over that of pristine graphene, especially for the individual ad-atom systems (compare curves d, c, and b to curve a in Supplementary Fig. 1b,c). The same trend is observed in experimental spectra (supplementary Fig. 1a), where a new low energy peak merges into the shoulder of the π-plasmon rise of graphene onto which Pd and Ti atoms were evaporated^39^. These new prominent peaks could be due to intraband transitions as predicted for doped graphene^40^,^41^,^42^,^43^, suggesting charge transfer between Pd and Ti atoms and graphene. Clusters of metal ad-atoms on graphene in accordance with^44^ also produce enhancement of at 3 eV (solid curves in Supplementary Fig. 2a,b) resulting in a broadening of the graphene π-plasmon. In contrast metal clusters alone show sharper transitions below the 4.7 eV feature (dashed curves in Supplementary Fig. 2a,b). As mentioned above, calculations for B- and N- (in-lattice-doped) CNTs show a similar pre-π-plasmon peak at 2.3–3 eV (Fig. 2a and Supplementary Fig. 4). As already mentioned, experimental spectra of B-doped CNTs exhibit a zero crossing of the R*ε*_*m*_, so do those of N-doped graphene (Fig. 2b); all this affirms the above suggestion of the creation of a charge carrier plasmon in the UV. The possibility of whether or not, however, the intraband transition created by ad-atoms or lattice dopants is coupled with single particle excitation in pristine graphene requires further investigations.

#### Local enhancement of UV spectral features at metal ad-atoms in graphene

**Figure 5:**
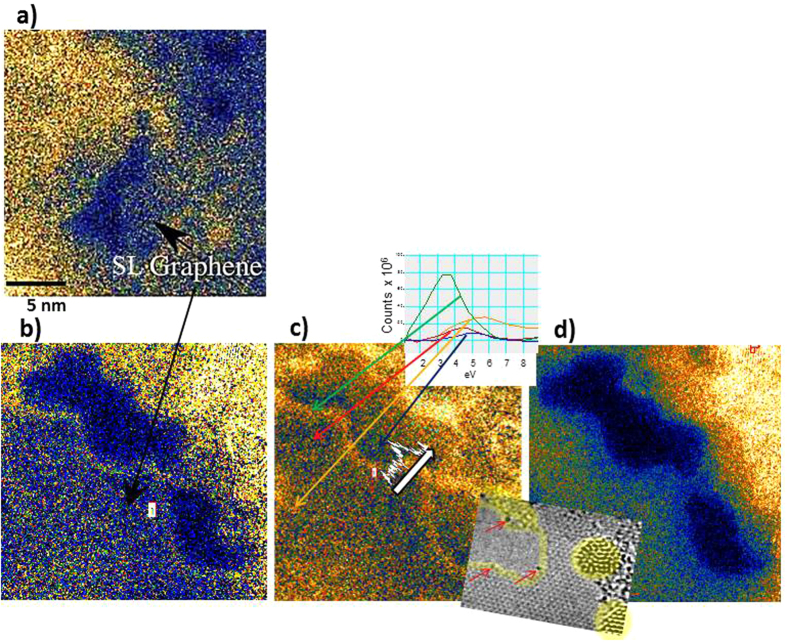
(**a,b**) EFTEM images from Fig. 4, showing the energy loss distribution at 3.5–4 eV in (**a**) pristine, un-doped graphene and (**b**) Pd-doped graphene, enlarged and displayed on the temperature scale. Dark blue colours signify low and yellow-white colours high intensity. The dark blue/ black regions are holes (vacuum), blue regions with a small amount of yellow signify single layer, clean graphene; an increase in the yellow ‘sprinkles’ on the graphene signifies thicker regions, due to hydro-carbon contamination, and bright yellow/white regions contain Pd-nanocrystals. Note the intensity enhancement at the holes in (**b**), and the lack of such enhancement in (**a**). (**c**) Loss intensity at 3.5–4 eV after additional subtraction of the π-plasmon background. Also shown in (**c**) is an intensity profile (white curve along arrow) across the graphene edge, and spectra extracted from various locations (green: edge of hole, red: clean part on SL graphene, yellow: contaminated region, blue: hole); the inset overlaid in the bottom right corner is an HREM image of that part of the sample, showing single Pd-atoms (arrowed) at the edge of the hole and Pd nano-crystals having formed on the SL graphene. The yellow sheen represents the plasmonic enhancement, (**d**) EFTEM image of same area as in (**b**) at the energy loss of 5–5.5 eV. Note the depletion in the intensity around the hole’s edge. *For details about samples and measurement see* Table 1.

We now focus on EFTEM studies of metal-dosed graphene, which demonstrate an enhancement of the ~3 eV absorption feature along the edges of holes in graphene, especially in the presence of Pd-atoms. Dosing with metal always results in non-uniform distributions. This is shown in the HREM image in Fig. 1c for the case of Pd. Rather than evenly covering the graphene, Pd forms nanoparticles of few nm in diameter. All nanoparticles sit on contamination (e.g., surrounding a patch of clean graphene in the middle in Fig. 1c) and do not reside on pristine graphene surfaces; neither have single Pd-atoms been detected on clean graphene patches. This is the case for all metals; a study of the metal-graphene interaction has previously been published^45^. In explanation of the observations theoretical works have predicted very low binding energies for metals on monolayer graphene^46^. The distributions and morphologies may vary somewhat for different metals, for example Ti (results not shown here) forms a large variety of cluster sizes down to the atomic level and is more dispersed than the Pd, but again, it sits mainly on/in the contamination on the graphene. EFTEM was used to carry out energy loss spectroscopy in the form of energy filtered imaging. In these experiments energy loss windows of between 0.4 and 1 eV were stepped with step sizes of 0.2 eV−0.5 eV from 17.5 eV to −2.5 eV, thus creating an EFTEM image series (data cube) over the specified energy range. A cube of corresponding dark reference images (a dark reference was taken directly after each energy loss image) was subtracted from the energy loss image data-cube. The exposure time in the energy region of the zero loss peak between −2 eV and 2 eV was 0.2 seconds (to avoid afterglow effects of the very intense spectral features in the low loss regime), whilst the exposure time for higher energies was 4 seconds. In the data cube, in which these images are compiled, the image plane coordinates are described by the x- and y-axes and the energy loss intensity is ascribed to coordinates along the z-axis, which represents an energy axis. The data assembly then represents a 4-dimensional data cube, and EEL spectra can be extracted along the z-axis for each x-y coordinate. The images in Fig. 4 (top row) are extracted from an EFTEM data cube of Pd-doped graphene. They show the change of the sample morphology during the recording, and, more importantly, the change in contrast exhibited by different structural features in image slices recorded at different loss energies. The EFTEM data cube, of which the images in Fig. 4 (top row) represent energy slices, was acquired from 17.5 eV to −2.5 eV in reverse mode, from high to low energy, to avoid afterglow effects from the high-intensity low loss region. The energy selecting slit width used here was 1 eV, the energy step size was 0.5 eV and the exposure time per slice was 5 seconds. The very bright (whitish) regions in Fig. 4 (top row), and also in Fig 5b–d (the latter are enlargements of the merged-hole region in Fig. 4, top row, 5^th^ image) represent Pd nanoparticles, whilst the darkest regions are holes in the graphene film. Example spectra from different locations, extracted from EFTEM cubes after subtraction of the zero loss peak are shown in Fig. 5c (inset overlay). In order to obtain the energy filtered image in Fig. 5c the excitation in the π-plasmon region at 4.5 eV was fitted by a Gaussian peak and subtracted from the data cube. The resulting EFTEM images highlight an excitation at ~3 eV in the various regions, including the edge of the hole in the pristine monolayer graphene. Although dynamic processes and changes in the morphology of the graphene were observed during prolonged exposure to the electron beam, EFTEM data cubes with sufficiently stable sample morphology could be obtained. The hole in Fig. 4 (top row) grew during exposure; however, in the energy regime of interest (below 7 eV) there was not much change as can be seen in the 4^th^ and 5^th^ image in Fig. 4, top row. Figure 5a,b are enlargements of the 5^th^ image in Fig. 4, top row. The HREM image inset overlay in Fig. 5c reveals individual Pd atoms (dark ‘dots’) at the edge along the hole in the graphene. Metals, e.g., Pd, Ti, and Si, have been reported to bind preferentially to edges of graphene sheets or to defect sites^46^. (STEM core loss spectroscopy of this sample, performed and published elsewhere^38^, confirmed the nature of the atoms, namely Pd and Ti, lining the edge of holes in graphene.) During imaging these atoms are observed to migrate along the hole’s edge. A significant enhancement in the energy loss can be seen at the Pd decorated edge in the UV/visible region of 3–4 eV in the EFTEM image in Fig. 5b. The enhancement is spatially confined to less than 0.5 nm along the edge of the sheet. The surfaces of the Pd nanoparticles also show significant energy loss at this energy (top of EFTEM image in Fig. 5a–c). This can be ascribed to the Pd surface plasmon. Line profiles of the integrated loss intensity in the 3–4 V energy regime across edges of holes (one of the line scan location is shown as white arrow in Fig. 5b, together with the corresponding intensity profile) show a relative enhancement over that of pristine graphene of up to 50%. Whilst enhancement of energy loss at metal adatoms in graphene^47^ and at pristine graphene edges^48^ has been reported before, this was only of the order of 1%. The here shown intense and highly localised enhancement has not been reported so far.

The origin of the enhancement is not known. It could be due to interband transitions affected by electronic structure changes of graphene due to the Pd atoms, or highly localised edge plasmons. There could also be occurrence of diffraction effects or edge folding/scrolling. Interestingly an energy-filtered image in the region of the π-plasmon energy, that is between 4.5 and 5 eV (Fig. 5d), shows a decrease in intensity near the edge of the graphene sheet. In order to confirm whether the enhancement is due to edge effects or due to the presence of the metal atoms, comparison to energy-loss properties of edges in pristine graphene was carried out. Figure 5a shows a slice (of the same energy as in Fig. 5b) from a ZLP-subtracted EFTEM image of pristine graphene. The localised enhancement in the Pd doped sample is significantly larger than any loss intensity in the respective energy regime at edges in the pristine samples; in fact the edge of the hole bordering clean graphene shows no enhancement, whereas the edge bordering clean graphene in the Pd dosed sample shows strong enhancement. This suggests that it is indeed the metal-atoms and not the electronic structure of graphene edges or folds that are the primary cause for the edge enhancement. The fact that the spectroscopic feature responsible for this enhancement is also found at the same energy after addition of various other dopants/adatoms indicates that it arises most likely due to the modification of the graphene bandstructure by these impurities.

## Conclusion

In a study combining high resolution electron microscopy imaging and energy loss spectroscopy we show that excitations in the 2–4 eV region are produced in nano-carbons, e.g., carbon nanotubes and graphene, upon doping and dosing with alkali and transition metals as well as group V and III elements. A new, distinct absorption peak at ~3 eV is introduced, which is most prominent in samples doped/dosed with K-, B-, N-, Ag- and Pd- atoms. These foreign atoms either adhere to the nanocarbons (on the surface or between atomic sheets) or are incorporated in the carbon lattice; they produce absorption features which comply with criteria for collective character, i.e, those for charge carrier plasmons arising by dopant induced Fermi level shifts in the nano-carbons, and not due to hybridization between the nano-carbons and the dopants. This is confirmed by observations and calculations of the real part of the dielectric function, which shows a zero crossing for the above dopants. However, more experiments as to the exact origin of this feature, and whether it is coupled to single particle excitations, are required. We furthermore demonstrate a localisation of this feature around the holes in edges of graphene dosed with metals (here Pd), which are preferred sites for metal atoms. This bears promise for the creation of plasmonic structures via nano-sculpting. A most noteworthy point to make is that the occurrence of this UV/vis feature at graphene edges conforms with k-vector requirements for coupling of plasmons to light, which would then be emitted from such edges, and vice versa. Hence graphene with nano-cavities, due to preferred metal-atom bonding to edges, is promising for incorporation as light emitters and detectors in nano-scale integrated photonic devices.

## Additional Information

**How to cite this article**: Bangert, U. *et al*. Collective electronic excitations in the ultra violet regime in 2-D and 1-D carbon nanostructures achieved by the addition of foreign atoms. *Sci. Rep.* **6**, 27090; doi: 10.1038/srep27090 (2016).

## Supplementary Material

Supplementary Information

## Figures and Tables

**Table 1:** Sample description.

**Dopant**	**Dose/Thickness**	**Introduced by**/**into**	**Investigated in**	**Shown in figure**
K	10^15^ cm^−2^	Ion implantation at 200 eV (Surrey Uni)/few-layer CNTs	Titan-Pico ER-C, FZ Juelich	1a, 2a, 2b
Ag	10^15^ cm^−2^	Ion implantation at 200 eV (Surrey Uni)/few-layer CNTs	SuperSTEM I, Daresbury, UK	1b, 2a, 2b
Na	10^15^ cm^−2^	Ion implantation at 200 eV (Surrey Uni)/few-layer CNTs	Titan-Pico ER-C, FZ Juelich	2a, 2b
B	10^15^ cm^−2^	Ion implantation at 200 eV (Surrey and Salford Uni)/few-layer CNTs	Liverpool NorthWest STEM	2a–d, 3a, 3c
Ba	10^15^ cm^−2^	Ion implantation at 200 eV (Surrey Uni)/few-layer CNTs	Titan-Pico ER-C, FZ Juelich	2a, 2b
N	10^15^ cm^−2^	Ion implantation at 200 eV (Surrey and Salford Uni)/few-layer CNTs	SuperSTEM I, Daresbury, UK	4 supplement
		Ion implantation at 25 eV (Goettingen Uni)/graphene	SuperSTEM II, Daresbury, UK	2a, 2b
Pd	0.1–0.3 nm	Evaporation/graphene	Titan-Pico ER-C, FZ Juelich	1c, 2a, 4 top row, 5b–c
Ti	0.1–0.3 nm	Evaporation/graphene	Titan-Pico ER-C, FZ Juelich	1a supplement
pristine	–	-/CNTs	Titan-Pico ER-C, FZ Juelich	2a, 2b, 3a–c, 4 supplement
		-/graphene	Titan-Pico ER-C, FZ Juelich	4 bottom row, 5a, 1a supplement
