# Supplementary material for: Collective electronic excitations in the ultra violet regime in 2-D and 1-D carbon nanostructures achieved by the addition of foreign atoms
Source: Sci Rep. 2016 Jun 7;6:27090. doi: 10.1038/srep27090 (PMC4917698; doi:10.1038/srep27090)
Supplement: Supplementary Information [file srep27090-s1.pdf]

Collective electronic excitations in the ultra violet regime in 2-D and 1-D carbon nanostructures achieved by the addition of foreign atoms

U. Bangert[1]*, W. Pierce[2], C. Boothroyd[3], C.-T. Pan[2], R. Gwilliam[4]

[1]Department of Physics and Energy, University of Limerick, Limerick, Ireland, *corresponding author
[2]School of Materials, The University of Manchester, Manchester M13 9PL, United Kingdom
[3]Ernst Ruska-Centre for Microscopy and Spectroscopy with Electrons and Peter Gruenberg Institute Juelich Research Centre, D-52425 Juelich, Germany
[4]Advanced Technology Institute, University of Surrey, Guildford GU2 7XH, United Kingdom

Supplementary Information

Density functional theory (DFT) methods [S1-S4] were used for modelling the electronic properties such as the total ground state energy, density of states and band structure as well as excited state properties including the optical absorption and dielectric function of graphene and carbon nanotubes. Two DFT codes, Wien2k and CASTEP, are used. Wien2k is an all-electron DFT code written in FORTRAN which runs on Unix-based systems [31, S5]. CASTEP is a DFT code based on the pseudopotential method [S6], which is available for UK and European academics via a license or internationally as part of the Accelrys Materials Studio. Both codes are capable of calculating the electronic properties of systems and both contain additional modules that allow the calculation of the low-loss and dielectric functions of EEL spectra [S7]. Convergence tests were carried out to find the minimum requirements for spectrum simulations in terms of K points and RK_{max}. The codes were applied to single and few-layer graphene and carbon nanotubes.

On a general note concerning parameters used in these calculations it has to be stated that for 2-D systems is it important to ensure that in there is no interaction between atomic layers (i.e., the z-dimension in repeat unit cells). This is done by having unit cells that represent sufficient vacuum spacing between the graphene sheets; the interlayer spacing between adjoining graphene layers was taken to be 40 Angstrom for all structures. Furthermore, supercell sizes of 2x2, 3x3 and 4x4, when used for complex structure arrangement, such ad-atom configurations on graphene, are relatively small (and thus the screening lengths might be insufficient); this can lead to artefacts, due to cross talk and interference of the atom impurity with itself. Hence results have to be treated with caution and awareness of such possible artefacts, especially concerning small-scale features/wiggles in the spectra.

Supplementary figure 1 shows calculated low loss spectra of graphene dosed with Pd and Ti for 2x2, 3x3 and 4x4 supercells, as well as experimental spectra, extracted from EFTEM image cubes, for comparison. Supplementary figure 2 shows calculated low loss spectra for metal atoms in various cluster configurations (shown on the left of (a)) on graphene. Supplementary figure 3 shows supercells of single wall carbon nanotubes with various doping levels and supplementary figure 4 shows Wien2K calculations of low loss spectra of N-doped CNTs

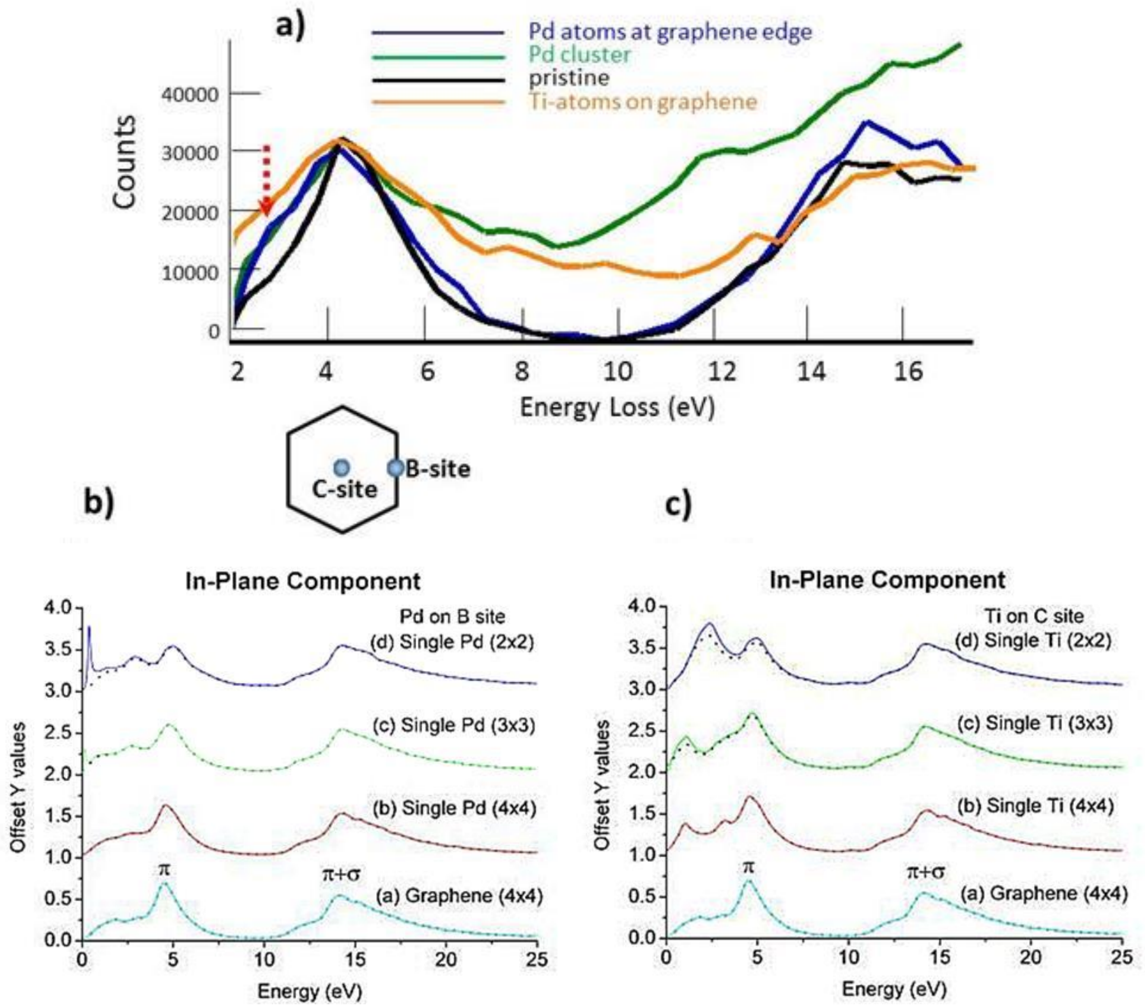

Supplementary Figure 1. a) low loss spectra of graphene dosed by evaporation with Pd and Ti, extracted from an EFTEM image cube.**b)** Simulated in-plane spectra of single Pd and **c)** of Ti adatoms on 2×2, 3×3 and 4×4 graphene supercells; the dashed curves are calculations without and the solid curves with intraband transitions.

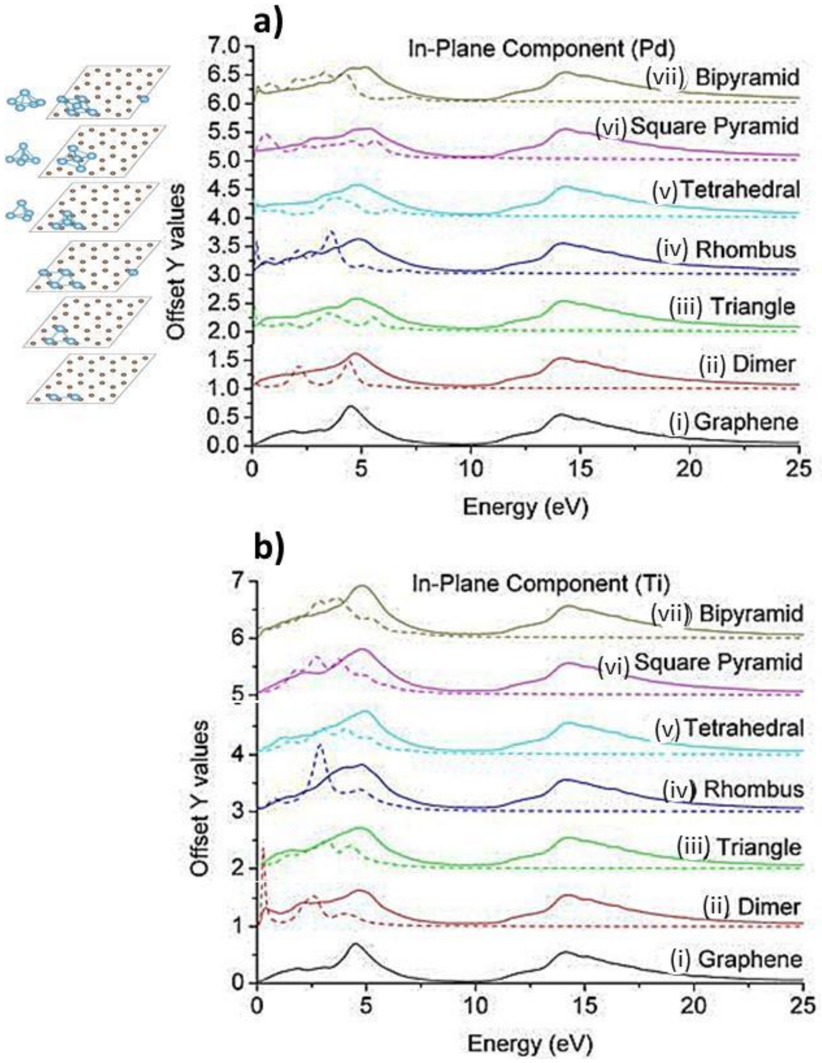

Supplementary Figure 2. Simulated in-plane EEL spectra (along the 'zig-zag' direction) of various Pd (a) and Ti (b) atom configurations (top left) on graphene; the spectra are of (i) pristine graphene, (ii) dimer, (iii) triangle, (iv) rhombus, (v) tetrahedra, (vi) square pyramid and (vii) bipyramid metal-clusters on graphene (solid curves), and of pure clusters without graphene (dashed curves). All spectra include intraband contributions.

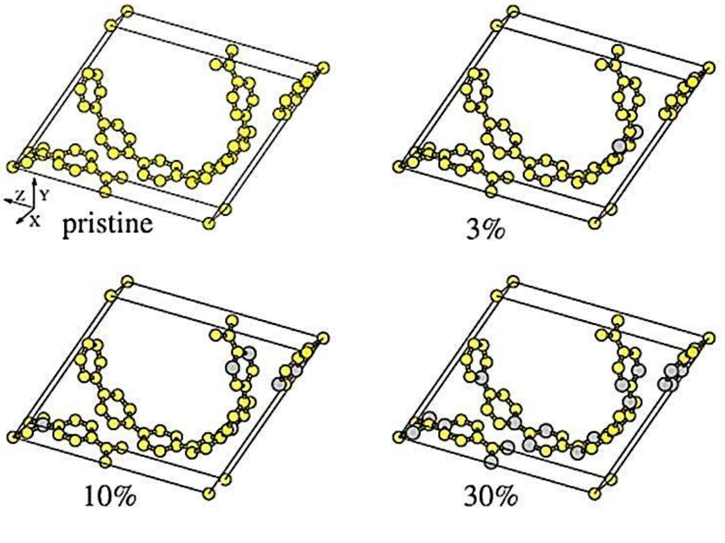

Supplementary Figure 3. Example SWNT models for various doping levels used in the loss function calculations. Carbon atoms are yellow, dopant atoms grey.

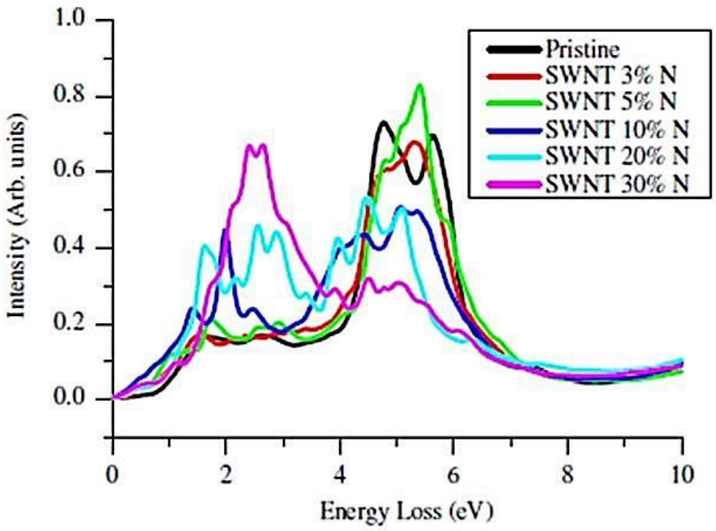

Supplementary Figure 4. Out-of-plane component of the low energy region of the loss function of N-doped (10, 10) SWNT bundles. Calculations were performed using Wien2K

References

S1. Scholl, D. S. & Steckel, J. A. *Density functional theory: a practical introduction* (Wiley, 2009).

S2. Cottenier, S. *Density functional theory and the family of (L)APW-methods: a step-by-step introduction*, 2nd ed. (2013) (http://www. wien2k.at/reguser/textbooks/DFT_andLAPW_2nd.pdf. Date of access:07/08/2013).

S3. Hohenberg, P. & Kohn, W. Inhomogeneous Electron Gas. *Phys. Rev*. **136,** B864–B871 (1964)

S4. Kohn, W. & Sham, L. J. Self-Consistent Equations Including Exchange and Correlation Effects. *Phys. Rev*. **140,** A1133–A1138 (1965).

S5. Schwarz, K., Blaha, P. & Madsen, G. Electronic structure calculations of solids using the WIEN2k package for material sciences.
Computer Physics Communications **147(1**–2), 71–76 (2002).
S6. Clark, S. J. *et al.* First principles methods using CASTEP. *Zeitschrift für Kristallographie* **220(5**–6), 567–570 (2005).
S7. Ambrosch-Draxl, C. & Sofo, J. O. Linear optical properties of solids within the full-potential linearized augmented planewave
method. *Comp. Phys. Communications* **175,** 1–14 (2006).
